# Zwitterionic form of tris­({[5-(4-meth­oxy­phenyl­azo)salicyl­idene]amino}­eth­yl)amine

**DOI:** 10.1107/S1600536811004405

**Published:** 2011-02-12

**Authors:** Sadegh Salehzadeh, Mahsa Mahdavian, Mehdi Khalaj

**Affiliations:** a Faculty of Chemistry, Bu-Ali Sina University, Hamedan, Iran; b Chemistry Department, Isalmic Azad University, Buinzahra Branch, Qazvin, Iran

## Abstract

The title compound (systematic name: 2-{[2-(bis­{2-[({2-hy­droxy-5-[(4-meth­oxy­phen­yl)diazen­yl]phen­yl}methyl­idene)amino]­eth­yl}amino)­eth­yl]aza­niumylidenemeth­yl}-4-[(4-meth­oxy­phen­yl)diazen­yl]phenolate), C_48_H_48_N_10_O_6_, exists as a zwitterion in the solid state. The three arms of the tripodal mol­ecule are located close to each other and an intra­molecular hydrogen bond occurs in each arm (O—H⋯N in two arms and N—H⋯O in the zwitterionic arm). The dihedral angles between the aromatic rings in the three arms are 16.36 (14), 23.94 (14) and, for the zwitterionic arm, 37.14 (14)°. In the crystal, a weak inter­moleclar N—H⋯O hydrogen bond occurs.

## Related literature

For tripodal Schiff base ligands, see: Kanesato *et al.* (2000 ▶) and for azo compounds, see: Butcher *et al.* (2005 ▶). For further synthetic details, see: Dinçalp *et al.* (2007 ▶).
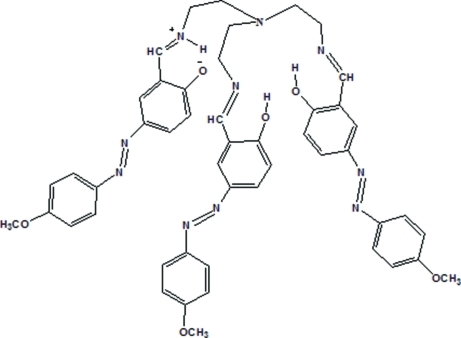


## Experimental

### 

#### Crystal data

C_48_H_48_N_10_O_6_
*M* *_r_* = 860.96
Triclinic, 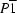

*a* = 10.5613 (9) Å
*b* = 12.1234 (5) Å
*c* = 17.2107 (9) Å
α = 86.418 (3)°
β = 89.308 (2)°
γ = 88.084 (3)°
*V* = 2198.0 (2) Å^3^
*Z* = 2
Mo *K*α radiation
μ = 0.09 mm^−1^
*T* = 150 K
0.22 × 0.20 × 0.16 mm

#### Data collection

Nonius KappaCCD diffractometer
Absorption correction: multi-scan (*SORTAV*; Blessing, 1995 ▶) *T*_min_ = 0.827, *T*_max_ = 0.990
19855 measured reflections
9781 independent reflections
4152 reflections with *I* > 2σ(*I*)
*R*_int_ = 0.071

#### Refinement

*R*[*F* ^2^ > 2σ(*F* ^2^)] = 0.062
*wR*(*F* ^2^) = 0.187
*S* = 0.99
9781 reflections
589 parameters
2 restraints
H atoms treated by a mixture of independent and constrained refinement
Δρ_max_ = 0.37 e Å^−3^
Δρ_min_ = −0.23 e Å^−3^

### 

Data collection: *COLLECT* (Nonius, 2002 ▶); cell refinement: *DENZO-SMN* (Otwinowski & Minor, 1997 ▶); data reduction: *DENZO-SMN*; program(s) used to solve structure: *SIR92* (Altomare *et al.*, 1994 ▶); program(s) used to refine structure: *SHELXTL* (Sheldrick, 2008 ▶); molecular graphics: *PLATON* (Spek, 2009 ▶); software used to prepare material for publication: *SHELXTL*.

## Supplementary Material

Crystal structure: contains datablocks I, global. DOI: 10.1107/S1600536811004405/hb5798sup1.cif

Structure factors: contains datablocks I. DOI: 10.1107/S1600536811004405/hb5798Isup2.hkl

Additional supplementary materials:  crystallographic information; 3D view; checkCIF report

## Figures and Tables

**Table 1:** Hydrogen-bond geometry (Å, °)

*D*—H⋯*A*	*D*—H	H⋯*A*	*D*⋯*A*	*D*—H⋯*A*
O3—H3⋯N4	1.07 (4)	1.58 (4)	2.543 (3)	146 (3)
O1—H1⋯N2	0.98 (4)	1.61 (4)	2.534 (3)	157 (3)
N3—H2⋯O2	1.02 (4)	1.71 (4)	2.585 (3)	142 (3)
N3—H2⋯O2^i^	1.02 (4)	2.48 (4)	3.155 (3)	123 (3)

Symmetry code: (i) 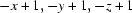.

**Fig. 1.**
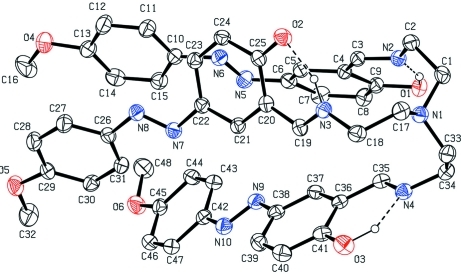
A view of the structure of (I), with displacement ellipsoids drawn at the 50% probability level. All C-bonded H atoms omitted for clarity.

## supplementary crystallographic information

### Comment

Herein, we report the synthesis and X-ray crystal structure of the title compound, (I), (Fig. 1), a tripodal Schiff base ligand containing three azo groups. For tripodal Schiff base ligands see: Kanesato *et al.* (2000) and for azo compounds see: Butcher *et al.* (2005).

The title compound adopts a cage-like conformation in the solid state. The geometry around the bridghead N atom of the compound is approximately pyramidal, since the angles C1–N1–C33, C1–N1–C17 and C33–N1–C17 have values of 112.3 (2), 113.5 (2) and 112.8 (2)°, respectively. The average of N═N bond lengths[1.25 (3) Å] is in the expected range and is in good agreement with values found in other similar compounds. Interestingly the hydrogen atom of one of three OH groups has transferred to one of three imine groups through intramolecular hydrogen bonding producing a zwitterionic compound. Three arms of tripodal ligand are located close to each other and in all of them there is an intramolecularhydrogen bonding (table 1).

### Experimental

Azo dye (5-(4-methoxyphenylazo)salicylaldehyde) was synthesized according to the literature procedure (Dinçalp *et al.*, 2007). Then to a solution of above aldehyde (3 mmol) in ethanol (70 ml) was added tren (1 mmol) in the same solvent (10 ml) (see Scheme I). The solution was stirred for 12 h at 40°C. The resulting orange precipitate was filtered and dried in vacuum. Orange blocks of (I) were obtained by slow evaporation from a acetonitril solution at room temperature after 24 h.

### Refinement

The H(C) atom positions were calculated and refined in isotropic approximatiom within riding model with the *U*_iso_(H) parameters equal to 1.2 *U*_eq_(Ci) where U(Ci) is the equivalent thermal parameters of the carbon atoms to which corresponding H atoms are bonded. 'H atoms bonded to C atoms were placed in calculated positions with C-H distances in the range 0.95-0.99 Å and were included in the refinement in a riding-model approximation with U_iso_(H) = 1.2U_eq_(C) or 1.5U_eq_(C_methyl_). H atoms bonded to O and N atoms were refined independently with isotropic displacement parameters.

### Crystal data

**Table:**

C_48_H_48_N_10_O_6_	*Z* = 2
*M**_r_* = 860.96	*F*(000) = 908
Triclinic, *P*1	*D*_x_ = 1.301 Mg m^−^^3^
Hall symbol: -P 1	Mo *K*α radiation, λ = 0.71073 Å
*a* = 10.5613 (9) Å	Cell parameters from 7794 reflections
*b* = 12.1234 (5) Å	θ = 2.6–27.5°
*c* = 17.2107 (9) Å	µ = 0.09 mm^−^^1^
α = 86.418 (3)°	*T* = 150 K
β = 89.308 (2)°	Block, orange
γ = 88.084 (3)°	0.22 × 0.20 × 0.16 mm
*V* = 2198.0 (2) Å^3^	

### Data collection

**Table:**

Nonius KappaCCD diffractometer	9781 independent reflections
Radiation source: fine-focus sealed tube	4152 reflections with *I* > 2σ(*I*)
graphite	*R*_int_ = 0.071
Detector resolution: 9 pixels mm^-1^	θ_max_ = 27.5°, θ_min_ = 2.6°
φ scans and ω scans with κ offsets	*h* = −13→13
Absorption correction: multi-scan (*SORTAV*; Blessing, 1995)	*k* = −15→15
*T*_min_ = 0.827, *T*_max_ = 0.990	*l* = −22→22
19855 measured reflections	

### Refinement

**Table:**

Refinement on *F*^2^	Primary atom site location: structure-invariant direct methods
Least-squares matrix: full	Secondary atom site location: difference Fourier map
*R*[*F*^2^ > 2σ(*F*^2^)] = 0.062	Hydrogen site location: inferred from neighbouring sites
*wR*(*F*^2^) = 0.187	H atoms treated by a mixture of independent and constrained refinement
*S* = 0.99	*w* = 1/[σ^2^(*F*_o_^2^) + (0.0768*P*)^2^] where *P* = (*F*_o_^2^ + 2*F*_c_^2^)/3
9781 reflections	(Δ/σ)_max_ = 0.001
589 parameters	Δρ_max_ = 0.37 e Å^−^^3^
2 restraints	Δρ_min_ = −0.23 e Å^−^^3^

### Special details

**Table:**

Geometry. All e.s.d.'s (except the e.s.d. in the dihedral angle between two l.s. planes) are estimated using the full covariance matrix. The cell e.s.d.'s are taken into account individually in the estimation of e.s.d.'s in distances, angles and torsion angles; correlations between e.s.d.'s in cell parameters are only used when they are defined by crystal symmetry. An approximate (isotropic) treatment of cell e.s.d.'s is used for estimating e.s.d.'s involving l.s. planes.
Refinement. Refinement of *F*^2^ against ALL reflections. The weighted *R*-factor *wR* and goodness of fit *S* are based on *F*^2^, conventional *R*-factors *R* are based on *F*, with *F* set to zero for negative *F*^2^. The threshold expression of *F*^2^ > σ(*F*^2^) is used only for calculating *R*-factors(gt) *etc*. and is not relevant to the choice of reflections for refinement. *R*-factors based on *F*^2^ are statistically about twice as large as those based on *F*, and *R*- factors based on ALL data will be even larger.

### Fractional atomic coordinates and isotropic or equivalent isotropic displacement parameters (Å^2^)

**Table:**

	*x*	*y*	*z*	*U*_iso_*/*U*_eq_	
O1	0.2448 (2)	0.82743 (16)	0.82789 (12)	0.0474 (6)	
O2	0.43622 (18)	0.45882 (17)	0.57762 (12)	0.0495 (6)	
O3	−0.0630 (2)	0.8117 (2)	0.50993 (12)	0.0576 (6)	
O4	−0.1109 (2)	−0.08081 (19)	0.91613 (15)	0.0776 (8)	
O5	−0.47340 (19)	−0.00203 (17)	0.79102 (13)	0.0582 (6)	
O6	−0.57085 (19)	0.29892 (18)	1.01599 (12)	0.0571 (6)	
N1	0.3726 (2)	0.8543 (2)	0.56017 (14)	0.0444 (6)	
N2	0.3882 (2)	0.74854 (19)	0.72451 (13)	0.0403 (6)	
N3	0.3183 (3)	0.6273 (2)	0.50901 (14)	0.0431 (6)	
N4	0.0959 (2)	0.8958 (2)	0.59704 (14)	0.0438 (6)	
N5	0.0284 (2)	0.4183 (2)	0.88438 (14)	0.0460 (6)	
N6	0.0856 (2)	0.3371 (2)	0.85796 (14)	0.0427 (6)	
N7	−0.0348 (2)	0.3043 (2)	0.66786 (13)	0.0418 (6)	
N8	−0.0348 (2)	0.2007 (2)	0.68282 (13)	0.0425 (6)	
N9	−0.2363 (2)	0.5682 (2)	0.77249 (14)	0.0457 (6)	
N10	−0.3386 (2)	0.5194 (2)	0.76315 (14)	0.0475 (7)	
C1	0.4720 (3)	0.8657 (2)	0.61690 (17)	0.0468 (8)	
H1A	0.4489	0.9278	0.6495	0.056*	
H1B	0.5521	0.8835	0.5891	0.056*	
C2	0.4926 (3)	0.7609 (2)	0.66887 (17)	0.0439 (8)	
H2A	0.4983	0.6961	0.6366	0.053*	
H2B	0.5733	0.7644	0.6972	0.053*	
C3	0.3336 (3)	0.6552 (2)	0.73432 (15)	0.0368 (7)	
H3A	0.3584	0.5963	0.7029	0.044*	
C4	0.2343 (2)	0.6388 (2)	0.79291 (15)	0.0355 (7)	
C5	0.1799 (3)	0.5360 (2)	0.80621 (16)	0.0393 (7)	
H5A	0.2052	0.4766	0.7754	0.047*	
C6	0.0884 (3)	0.5204 (3)	0.86465 (17)	0.0422 (7)	
C7	0.0480 (3)	0.6087 (3)	0.90776 (17)	0.0476 (8)	
H7A	−0.0164	0.5984	0.9463	0.057*	
C8	0.1006 (3)	0.7108 (3)	0.89495 (16)	0.0459 (8)	
H8A	0.0728	0.7705	0.9248	0.055*	
C9	0.1939 (3)	0.7264 (2)	0.83858 (16)	0.0384 (7)	
C10	0.0247 (3)	0.2334 (2)	0.87651 (16)	0.0405 (7)	
C11	0.0921 (3)	0.1410 (3)	0.85677 (18)	0.0494 (8)	
H11A	0.1733	0.1485	0.8330	0.059*	
C12	0.0447 (3)	0.0371 (3)	0.87057 (19)	0.0574 (9)	
H12A	0.0926	−0.0264	0.8564	0.069*	
C13	−0.0730 (3)	0.0262 (3)	0.90515 (19)	0.0508 (8)	
C14	−0.1426 (3)	0.1186 (3)	0.92557 (17)	0.0520 (9)	
H14A	−0.2237	0.1114	0.9495	0.062*	
C15	−0.0925 (3)	0.2223 (3)	0.91061 (16)	0.0468 (8)	
H15A	−0.1399	0.2863	0.9242	0.056*	
C16	−0.2313 (4)	−0.0982 (3)	0.9522 (2)	0.0927 (15)	
H16A	−0.2475	−0.1775	0.9563	0.139*	
H16B	−0.2974	−0.0590	0.9208	0.139*	
H16C	−0.2318	−0.0701	1.0044	0.139*	
C17	0.4180 (3)	0.8058 (3)	0.48855 (17)	0.0487 (8)	
H17A	0.4997	0.7654	0.4989	0.058*	
H17B	0.4333	0.8659	0.4482	0.058*	
C18	0.3250 (3)	0.7275 (2)	0.45807 (17)	0.0489 (8)	
H18A	0.2401	0.7645	0.4544	0.059*	
H18B	0.3515	0.7080	0.4052	0.059*	
C19	0.2148 (3)	0.5907 (2)	0.54053 (16)	0.0422 (7)	
H19A	0.1382	0.6330	0.5325	0.051*	
C20	0.2118 (3)	0.4892 (2)	0.58677 (16)	0.0391 (7)	
C21	0.0946 (3)	0.4468 (2)	0.61236 (16)	0.0392 (7)	
H21A	0.0193	0.4910	0.6044	0.047*	
C22	0.0871 (3)	0.3435 (2)	0.64833 (16)	0.0397 (7)	
C23	0.2004 (3)	0.2813 (2)	0.66447 (16)	0.0411 (7)	
H23A	0.1959	0.2101	0.6906	0.049*	
C24	0.3155 (3)	0.3206 (2)	0.64355 (15)	0.0413 (7)	
H24A	0.3897	0.2777	0.6573	0.050*	
C25	0.3276 (3)	0.4250 (2)	0.60136 (16)	0.0393 (7)	
C26	−0.1541 (3)	0.1589 (2)	0.70770 (16)	0.0404 (7)	
C27	−0.1660 (3)	0.0458 (3)	0.7050 (2)	0.0548 (9)	
H27A	−0.0983	0.0027	0.6844	0.066*	
C28	−0.2738 (3)	−0.0052 (3)	0.7316 (2)	0.0573 (9)	
H28A	−0.2810	−0.0826	0.7283	0.069*	
C29	−0.3711 (3)	0.0563 (3)	0.76274 (18)	0.0453 (8)	
C30	−0.3613 (3)	0.1697 (2)	0.76609 (16)	0.0430 (8)	
H30A	−0.4288	0.2125	0.7873	0.052*	
C31	−0.2526 (3)	0.2202 (2)	0.73831 (16)	0.0413 (7)	
H31A	−0.2459	0.2979	0.7404	0.050*	
C32	−0.5733 (3)	0.0585 (3)	0.8261 (2)	0.0722 (11)	
H32A	−0.6401	0.0081	0.8435	0.108*	
H32B	−0.6081	0.1147	0.7881	0.108*	
H32C	−0.5410	0.0945	0.8709	0.108*	
C33	0.2976 (3)	0.9573 (3)	0.54508 (19)	0.0515 (9)	
H33A	0.2576	0.9564	0.4934	0.062*	
H33B	0.3547	1.0206	0.5436	0.062*	
C34	0.1956 (3)	0.9737 (3)	0.60625 (18)	0.0497 (8)	
H34A	0.2325	0.9617	0.6588	0.060*	
H34B	0.1600	1.0503	0.6006	0.060*	
C35	0.0569 (3)	0.8342 (2)	0.65497 (18)	0.0420 (7)	
H35A	0.0957	0.8376	0.7042	0.050*	
C36	−0.0453 (3)	0.7596 (2)	0.64655 (17)	0.0379 (7)	
C37	−0.0930 (3)	0.6951 (2)	0.71012 (17)	0.0413 (7)	
H37A	−0.0565	0.7003	0.7599	0.050*	
C38	−0.1911 (3)	0.6244 (2)	0.70259 (17)	0.0412 (7)	
C39	−0.2429 (3)	0.6148 (3)	0.62893 (18)	0.0484 (8)	
H39A	−0.3081	0.5641	0.6225	0.058*	
C40	−0.1999 (3)	0.6784 (3)	0.56589 (18)	0.0491 (8)	
H40A	−0.2370	0.6725	0.5164	0.059*	
C41	−0.1031 (3)	0.7508 (3)	0.57366 (17)	0.0436 (8)	
C42	−0.3878 (3)	0.4641 (2)	0.83188 (18)	0.0424 (7)	
C43	−0.3285 (3)	0.4523 (2)	0.90327 (18)	0.0476 (8)	
H43A	−0.2474	0.4822	0.9091	0.057*	
C44	−0.3864 (3)	0.3974 (2)	0.96617 (19)	0.0484 (8)	
H44A	−0.3451	0.3892	1.0150	0.058*	
C45	−0.5053 (3)	0.3540 (2)	0.95763 (18)	0.0437 (8)	
C46	−0.5658 (3)	0.3664 (2)	0.88634 (18)	0.0466 (8)	
H46A	−0.6480	0.3384	0.8807	0.056*	
C47	−0.5060 (3)	0.4197 (2)	0.82372 (18)	0.0445 (8)	
H47A	−0.5461	0.4260	0.7745	0.053*	
C48	−0.5098 (3)	0.2782 (3)	1.08894 (19)	0.0662 (10)	
H48A	−0.5668	0.2386	1.1255	0.099*	
H48B	−0.4884	0.3486	1.1097	0.099*	
H48C	−0.4322	0.2331	1.0819	0.099*	
H1	0.308 (4)	0.816 (3)	0.787 (2)	0.103 (14)*	
H2	0.392 (4)	0.573 (3)	0.519 (2)	0.100 (14)*	
H3	0.005 (4)	0.867 (3)	0.529 (2)	0.103 (13)*	

### Atomic displacement parameters (Å^2^)

**Table:**

	*U*^11^	*U*^22^	*U*^33^	*U*^12^	*U*^13^	*U*^23^
O1	0.0573 (14)	0.0363 (13)	0.0490 (14)	−0.0036 (11)	0.0010 (11)	−0.0046 (10)
O2	0.0392 (12)	0.0557 (14)	0.0529 (13)	−0.0072 (11)	0.0028 (10)	0.0031 (11)
O3	0.0633 (15)	0.0720 (16)	0.0368 (13)	−0.0080 (13)	−0.0009 (11)	0.0047 (11)
O4	0.0704 (17)	0.0500 (15)	0.110 (2)	−0.0222 (14)	−0.0129 (15)	0.0250 (14)
O5	0.0429 (13)	0.0481 (14)	0.0844 (17)	−0.0111 (11)	0.0115 (12)	−0.0067 (12)
O6	0.0515 (13)	0.0604 (15)	0.0587 (15)	−0.0159 (12)	−0.0064 (11)	0.0113 (12)
N1	0.0423 (14)	0.0430 (16)	0.0467 (16)	−0.0039 (13)	0.0005 (12)	0.0069 (12)
N2	0.0393 (14)	0.0391 (15)	0.0419 (15)	−0.0059 (12)	−0.0013 (11)	0.0052 (12)
N3	0.0450 (16)	0.0394 (16)	0.0443 (16)	−0.0041 (14)	−0.0011 (12)	0.0046 (12)
N4	0.0435 (15)	0.0435 (16)	0.0435 (16)	−0.0001 (13)	0.0017 (12)	0.0037 (13)
N5	0.0510 (16)	0.0430 (15)	0.0435 (16)	−0.0004 (13)	0.0029 (12)	0.0005 (13)
N6	0.0430 (14)	0.0414 (16)	0.0436 (16)	0.0015 (13)	−0.0007 (12)	−0.0027 (13)
N7	0.0406 (15)	0.0424 (16)	0.0421 (15)	−0.0036 (13)	−0.0012 (11)	0.0010 (12)
N8	0.0380 (14)	0.0400 (16)	0.0494 (16)	−0.0053 (12)	0.0022 (11)	0.0001 (12)
N9	0.0362 (14)	0.0437 (16)	0.0563 (17)	0.0001 (13)	0.0005 (12)	0.0020 (13)
N10	0.0423 (15)	0.0434 (16)	0.0563 (17)	−0.0005 (13)	0.0014 (13)	−0.0011 (13)
C1	0.0449 (18)	0.0451 (19)	0.0500 (19)	−0.0146 (16)	0.0008 (15)	0.0068 (15)
C2	0.0349 (16)	0.0456 (19)	0.0502 (19)	−0.0052 (15)	0.0028 (14)	0.0069 (15)
C3	0.0391 (16)	0.0346 (17)	0.0362 (17)	−0.0026 (14)	−0.0023 (13)	0.0011 (13)
C4	0.0361 (16)	0.0338 (17)	0.0363 (17)	−0.0036 (14)	−0.0021 (13)	0.0022 (14)
C5	0.0417 (17)	0.0373 (18)	0.0386 (17)	−0.0049 (14)	−0.0070 (14)	0.0043 (14)
C6	0.0416 (17)	0.0457 (18)	0.0383 (17)	−0.0076 (15)	−0.0042 (14)	0.0091 (15)
C7	0.0518 (19)	0.053 (2)	0.0372 (18)	0.0022 (17)	0.0066 (15)	0.0029 (16)
C8	0.058 (2)	0.0410 (19)	0.0384 (18)	−0.0017 (16)	0.0006 (15)	−0.0017 (15)
C9	0.0428 (17)	0.0359 (18)	0.0363 (17)	−0.0045 (15)	−0.0029 (14)	0.0027 (14)
C10	0.0433 (18)	0.0397 (19)	0.0377 (18)	−0.0064 (16)	−0.0026 (14)	0.0071 (14)
C11	0.0462 (19)	0.043 (2)	0.058 (2)	0.0001 (17)	0.0081 (16)	−0.0002 (16)
C12	0.060 (2)	0.037 (2)	0.075 (2)	−0.0009 (17)	0.0048 (19)	−0.0001 (17)
C13	0.054 (2)	0.043 (2)	0.054 (2)	−0.0083 (18)	−0.0071 (16)	0.0164 (16)
C14	0.0422 (18)	0.068 (2)	0.044 (2)	−0.0058 (19)	0.0040 (15)	0.0052 (17)
C15	0.0526 (19)	0.045 (2)	0.0428 (19)	−0.0007 (16)	−0.0025 (15)	−0.0013 (15)
C16	0.070 (3)	0.095 (3)	0.110 (3)	−0.047 (3)	−0.019 (2)	0.045 (3)
C17	0.0464 (18)	0.052 (2)	0.046 (2)	−0.0053 (16)	0.0106 (15)	0.0057 (16)
C18	0.0484 (18)	0.053 (2)	0.0441 (19)	−0.0024 (17)	−0.0008 (15)	0.0067 (16)
C19	0.0408 (17)	0.0424 (17)	0.0440 (18)	−0.0059 (15)	0.0021 (14)	−0.0053 (13)
C20	0.0423 (17)	0.0392 (16)	0.0362 (17)	−0.0047 (15)	0.0038 (13)	−0.0053 (12)
C21	0.0374 (17)	0.0362 (18)	0.0443 (18)	0.0005 (14)	0.0031 (13)	−0.0066 (14)
C22	0.0361 (17)	0.0387 (18)	0.0442 (18)	−0.0038 (15)	0.0024 (14)	−0.0021 (14)
C23	0.0442 (18)	0.0394 (18)	0.0398 (18)	−0.0033 (15)	−0.0009 (14)	−0.0015 (14)
C24	0.0404 (17)	0.0441 (19)	0.0394 (18)	0.0001 (15)	−0.0052 (14)	−0.0016 (15)
C25	0.0413 (18)	0.0425 (19)	0.0350 (17)	−0.0046 (15)	−0.0008 (13)	−0.0070 (14)
C26	0.0352 (17)	0.0413 (19)	0.0447 (18)	−0.0060 (15)	0.0000 (14)	0.0003 (15)
C27	0.0396 (18)	0.041 (2)	0.084 (3)	−0.0007 (16)	0.0092 (17)	−0.0078 (18)
C28	0.0412 (19)	0.040 (2)	0.092 (3)	−0.0058 (17)	0.0081 (18)	−0.0106 (18)
C29	0.0367 (17)	0.043 (2)	0.056 (2)	−0.0108 (16)	0.0003 (15)	−0.0011 (16)
C30	0.0397 (17)	0.0408 (19)	0.0478 (19)	0.0021 (15)	0.0069 (14)	−0.0010 (15)
C31	0.0443 (18)	0.0352 (17)	0.0441 (18)	−0.0046 (15)	0.0001 (14)	0.0022 (14)
C32	0.054 (2)	0.062 (3)	0.100 (3)	−0.010 (2)	0.029 (2)	−0.003 (2)
C33	0.052 (2)	0.044 (2)	0.057 (2)	−0.0073 (17)	−0.0002 (16)	0.0115 (16)
C34	0.0513 (19)	0.0378 (19)	0.059 (2)	0.0016 (16)	−0.0034 (16)	0.0024 (16)
C35	0.0391 (17)	0.0427 (19)	0.0432 (19)	0.0066 (15)	−0.0024 (14)	0.0002 (15)
C36	0.0352 (16)	0.0368 (17)	0.0410 (18)	0.0071 (14)	0.0008 (13)	−0.0004 (14)
C37	0.0365 (16)	0.0456 (19)	0.0416 (18)	0.0012 (15)	−0.0070 (13)	0.0000 (15)
C38	0.0345 (16)	0.0414 (18)	0.0465 (19)	0.0064 (15)	0.0035 (14)	0.0016 (15)
C39	0.0434 (18)	0.048 (2)	0.055 (2)	−0.0021 (16)	0.0027 (16)	−0.0147 (17)
C40	0.0484 (19)	0.061 (2)	0.0383 (18)	0.0025 (17)	−0.0013 (15)	−0.0097 (16)
C41	0.0406 (17)	0.050 (2)	0.0397 (19)	0.0070 (16)	0.0021 (14)	−0.0007 (15)
C42	0.0376 (17)	0.0347 (18)	0.054 (2)	0.0017 (14)	0.0000 (15)	0.0007 (15)
C43	0.0365 (17)	0.0442 (19)	0.062 (2)	0.0005 (15)	−0.0075 (16)	0.0000 (16)
C44	0.0391 (18)	0.051 (2)	0.055 (2)	−0.0069 (16)	−0.0086 (15)	0.0059 (16)
C45	0.0391 (17)	0.0350 (18)	0.057 (2)	−0.0041 (15)	0.0034 (15)	0.0004 (15)
C46	0.0372 (17)	0.0437 (19)	0.059 (2)	−0.0045 (15)	−0.0053 (16)	−0.0011 (16)
C47	0.0399 (17)	0.0423 (19)	0.051 (2)	0.0005 (15)	−0.0059 (15)	−0.0035 (15)
C48	0.066 (2)	0.072 (3)	0.060 (2)	−0.018 (2)	−0.0120 (19)	0.0141 (19)

### Geometric parameters (Å, °)

**Table:**

O1—C9	1.355 (3)	C17—C18	1.509 (4)
O1—H1	0.98 (4)	C17—H17A	0.9900
O2—C25	1.284 (3)	C17—H17B	0.9900
O3—C41	1.355 (3)	C18—H18A	0.9900
O3—H3	1.07 (4)	C18—H18B	0.9900
O4—C13	1.371 (3)	C19—C20	1.424 (4)
O4—C16	1.425 (4)	C19—H19A	0.9500
O5—C29	1.378 (3)	C20—C21	1.411 (4)
O5—C32	1.416 (4)	C20—C25	1.444 (4)
O6—C45	1.366 (3)	C21—C22	1.366 (4)
O6—C48	1.423 (3)	C21—H21A	0.9500
N1—C1	1.459 (3)	C22—C23	1.414 (4)
N1—C33	1.467 (4)	C23—C24	1.357 (4)
N1—C17	1.467 (4)	C23—H23A	0.9500
N2—C3	1.288 (3)	C24—C25	1.428 (4)
N2—C2	1.457 (3)	C24—H24A	0.9500
N3—C19	1.294 (3)	C26—C31	1.377 (4)
N3—C18	1.457 (4)	C26—C27	1.385 (4)
N3—H2	1.02 (4)	C27—C28	1.373 (4)
N4—C35	1.281 (3)	C27—H27A	0.9500
N4—C34	1.454 (3)	C28—C29	1.372 (4)
N5—N6	1.245 (3)	C28—H28A	0.9500
N5—C6	1.429 (3)	C29—C30	1.388 (4)
N6—C10	1.447 (3)	C30—C31	1.385 (4)
N7—N8	1.266 (3)	C30—H30A	0.9500
N7—C22	1.417 (3)	C31—H31A	0.9500
N8—C26	1.425 (3)	C32—H32A	0.9800
N9—N10	1.266 (3)	C32—H32B	0.9800
N9—C38	1.430 (4)	C32—H32C	0.9800
N10—C42	1.424 (4)	C33—C34	1.514 (4)
C1—C2	1.518 (4)	C33—H33A	0.9900
C1—H1A	0.9900	C33—H33B	0.9900
C1—H1B	0.9900	C34—H34A	0.9900
C2—H2A	0.9900	C34—H34B	0.9900
C2—H2B	0.9900	C35—C36	1.445 (4)
C3—C4	1.456 (4)	C35—H35A	0.9500
C3—H3A	0.9500	C36—C37	1.403 (4)
C4—C5	1.395 (3)	C36—C41	1.414 (4)
C4—C9	1.412 (4)	C37—C38	1.380 (4)
C5—C6	1.395 (4)	C37—H37A	0.9500
C5—H5A	0.9500	C38—C39	1.400 (4)
C6—C7	1.393 (4)	C39—C40	1.373 (4)
C7—C8	1.378 (4)	C39—H39A	0.9500
C7—H7A	0.9500	C40—C41	1.382 (4)
C8—C9	1.384 (4)	C40—H40A	0.9500
C8—H8A	0.9500	C42—C43	1.383 (4)
C10—C11	1.366 (4)	C42—C47	1.388 (4)
C10—C15	1.371 (4)	C43—C44	1.382 (4)
C11—C12	1.376 (4)	C43—H43A	0.9500
C11—H11A	0.9500	C44—C45	1.390 (4)
C12—C13	1.379 (4)	C44—H44A	0.9500
C12—H12A	0.9500	C45—C46	1.388 (4)
C13—C14	1.381 (4)	C46—C47	1.379 (4)
C14—C15	1.388 (4)	C46—H46A	0.9500
C14—H14A	0.9500	C47—H47A	0.9500
C15—H15A	0.9500	C48—H48A	0.9800
C16—H16A	0.9800	C48—H48B	0.9800
C16—H16B	0.9800	C48—H48C	0.9800
C16—H16C	0.9800		
			
C9—O1—H1	102 (2)	C22—C21—H21A	119.3
C41—O3—H3	107 (2)	C20—C21—H21A	119.3
C13—O4—C16	117.4 (3)	C21—C22—C23	118.8 (3)
C29—O5—C32	117.3 (2)	C21—C22—N7	117.9 (3)
C45—O6—C48	117.7 (2)	C23—C22—N7	123.2 (3)
C1—N1—C33	112.3 (2)	C24—C23—C22	121.7 (3)
C1—N1—C17	113.5 (2)	C24—C23—H23A	119.2
C33—N1—C17	112.8 (2)	C22—C23—H23A	119.2
C3—N2—C2	119.8 (3)	C23—C24—C25	121.5 (3)
C19—N3—C18	124.2 (3)	C23—C24—H24A	119.2
C19—N3—H2	111 (2)	C25—C24—H24A	119.2
C18—N3—H2	124 (2)	O2—C25—C24	121.2 (3)
C35—N4—C34	120.9 (3)	O2—C25—C20	122.4 (3)
N6—N5—C6	113.1 (2)	C24—C25—C20	116.4 (3)
N5—N6—C10	113.6 (2)	C31—C26—C27	118.7 (3)
N8—N7—C22	113.0 (3)	C31—C26—N8	125.4 (3)
N7—N8—C26	114.7 (3)	C27—C26—N8	115.8 (3)
N10—N9—C38	113.0 (2)	C28—C27—C26	121.3 (3)
N9—N10—C42	114.6 (2)	C28—C27—H27A	119.4
N1—C1—C2	111.9 (2)	C26—C27—H27A	119.4
N1—C1—H1A	109.2	C29—C28—C27	119.7 (3)
C2—C1—H1A	109.2	C29—C28—H28A	120.1
N1—C1—H1B	109.2	C27—C28—H28A	120.1
C2—C1—H1B	109.2	C28—C29—O5	116.0 (3)
H1A—C1—H1B	107.9	C28—C29—C30	120.1 (3)
N2—C2—C1	110.1 (2)	O5—C29—C30	123.9 (3)
N2—C2—H2A	109.6	C31—C30—C29	119.6 (3)
C1—C2—H2A	109.6	C31—C30—H30A	120.2
N2—C2—H2B	109.6	C29—C30—H30A	120.2
C1—C2—H2B	109.6	C26—C31—C30	120.7 (3)
H2A—C2—H2B	108.2	C26—C31—H31A	119.7
N2—C3—C4	120.8 (3)	C30—C31—H31A	119.7
N2—C3—H3A	119.6	O5—C32—H32A	109.5
C4—C3—H3A	119.6	O5—C32—H32B	109.5
C5—C4—C9	119.0 (2)	H32A—C32—H32B	109.5
C5—C4—C3	120.7 (3)	O5—C32—H32C	109.5
C9—C4—C3	120.3 (2)	H32A—C32—H32C	109.5
C4—C5—C6	120.1 (3)	H32B—C32—H32C	109.5
C4—C5—H5A	120.0	N1—C33—C34	112.5 (2)
C6—C5—H5A	120.0	N1—C33—H33A	109.1
C7—C6—C5	119.9 (3)	C34—C33—H33A	109.1
C7—C6—N5	115.3 (3)	N1—C33—H33B	109.1
C5—C6—N5	124.9 (3)	C34—C33—H33B	109.1
C8—C7—C6	120.6 (3)	H33A—C33—H33B	107.8
C8—C7—H7A	119.7	N4—C34—C33	109.4 (2)
C6—C7—H7A	119.7	N4—C34—H34A	109.8
C7—C8—C9	120.0 (3)	C33—C34—H34A	109.8
C7—C8—H8A	120.0	N4—C34—H34B	109.8
C9—C8—H8A	120.0	C33—C34—H34B	109.8
O1—C9—C8	118.7 (3)	H34A—C34—H34B	108.2
O1—C9—C4	120.9 (2)	N4—C35—C36	121.0 (3)
C8—C9—C4	120.5 (3)	N4—C35—H35A	119.5
C11—C10—C15	119.3 (3)	C36—C35—H35A	119.5
C11—C10—N6	115.4 (3)	C37—C36—C41	117.3 (3)
C15—C10—N6	125.4 (3)	C37—C36—C35	121.8 (3)
C10—C11—C12	121.3 (3)	C41—C36—C35	120.9 (3)
C10—C11—H11A	119.3	C38—C37—C36	122.0 (3)
C12—C11—H11A	119.3	C38—C37—H37A	119.0
C11—C12—C13	119.3 (3)	C36—C37—H37A	119.0
C11—C12—H12A	120.4	C37—C38—C39	119.0 (3)
C13—C12—H12A	120.4	C37—C38—N9	116.7 (3)
O4—C13—C12	114.4 (3)	C39—C38—N9	124.2 (3)
O4—C13—C14	125.3 (3)	C40—C39—C38	120.3 (3)
C12—C13—C14	120.3 (3)	C40—C39—H39A	119.9
C13—C14—C15	119.1 (3)	C38—C39—H39A	119.9
C13—C14—H14A	120.5	C39—C40—C41	120.7 (3)
C15—C14—H14A	120.5	C39—C40—H40A	119.6
C10—C15—C14	120.8 (3)	C41—C40—H40A	119.6
C10—C15—H15A	119.6	O3—C41—C40	118.9 (3)
C14—C15—H15A	119.6	O3—C41—C36	120.5 (3)
O4—C16—H16A	109.5	C40—C41—C36	120.6 (3)
O4—C16—H16B	109.5	C43—C42—C47	119.4 (3)
H16A—C16—H16B	109.5	C43—C42—N10	125.8 (3)
O4—C16—H16C	109.5	C47—C42—N10	114.8 (3)
H16A—C16—H16C	109.5	C44—C43—C42	120.5 (3)
H16B—C16—H16C	109.5	C44—C43—H43A	119.8
N1—C17—C18	112.3 (2)	C42—C43—H43A	119.8
N1—C17—H17A	109.1	C43—C44—C45	119.7 (3)
C18—C17—H17A	109.1	C43—C44—H44A	120.1
N1—C17—H17B	109.1	C45—C44—H44A	120.1
C18—C17—H17B	109.1	O6—C45—C46	115.5 (2)
H17A—C17—H17B	107.9	O6—C45—C44	124.4 (3)
N3—C18—C17	110.9 (2)	C46—C45—C44	120.1 (3)
N3—C18—H18A	109.5	C47—C46—C45	119.5 (3)
C17—C18—H18A	109.5	C47—C46—H46A	120.2
N3—C18—H18B	109.5	C45—C46—H46A	120.2
C17—C18—H18B	109.5	C46—C47—C42	120.7 (3)
H18A—C18—H18B	108.1	C46—C47—H47A	119.6
N3—C19—C20	122.2 (3)	C42—C47—H47A	119.6
N3—C19—H19A	118.9	O6—C48—H48A	109.5
C20—C19—H19A	118.9	O6—C48—H48B	109.5
C21—C20—C19	120.0 (3)	H48A—C48—H48B	109.5
C21—C20—C25	120.0 (3)	O6—C48—H48C	109.5
C19—C20—C25	119.8 (3)	H48A—C48—H48C	109.5
C22—C21—C20	121.4 (3)	H48B—C48—H48C	109.5

### Hydrogen-bond geometry (Å, °)

**Table:**

*D*—H···*A*	*D*—H	H···*A*	*D*···*A*	*D*—H···*A*
O3—H3···N4	1.07 (4)	1.58 (4)	2.543 (3)	146 (3)
O1—H1···N2	0.98 (4)	1.61 (4)	2.534 (3)	157 (3)
N3—H2···O2	1.02 (4)	1.71 (4)	2.585 (3)	142 (3)
N3—H2···O2^i^	1.02 (4)	2.48 (4)	3.155 (3)	123 (3)

Symmetry codes: (i) −*x*+1, −*y*+1, −*z*+1.
